# Codeveloping and Evaluating a Campaign to Reduce Dementia Misconceptions on Twitter: Machine Learning Study

**DOI:** 10.2196/36871

**Published:** 2022-11-22

**Authors:** Sinan Erturk, Georgie Hudson, Sonja M Jansli, Daniel Morris, Clarissa M Odoi, Emma Wilson, Angela Clayton-Turner, Vanessa Bray, Gill Yourston, Andrew Cornwall, Nicholas Cummins, Til Wykes, Sagar Jilka

**Affiliations:** 1 Institute of Psychiatry, Psychology & Neuroscience King's College London London United Kingdom; 2 South London and Maudsley NHS Foundation Trust London United Kingdom; 3 Warwick Medical School University of Warwick Coventry United Kingdom

**Keywords:** machine learning, patient and public involvement, codevelopment, misconceptions, stigma, Twitter, social media

## Abstract

**Background:**

Dementia misconceptions on Twitter can have detrimental or harmful effects. Machine learning (ML) models codeveloped with carers provide a method to identify these and help in evaluating awareness campaigns.

**Objective:**

This study aimed to develop an ML model to distinguish between misconceptions and neutral tweets and to develop, deploy, and evaluate an awareness campaign to tackle dementia misconceptions.

**Methods:**

Taking 1414 tweets rated by carers from our previous work, we built 4 ML models. Using a 5-fold cross-validation, we evaluated them and performed a further blind validation with carers for the best 2 ML models; from this blind validation, we selected the best model overall. We codeveloped an awareness campaign and collected pre-post campaign tweets (N=4880), classifying them with our model as misconceptions or not. We analyzed dementia tweets from the United Kingdom across the campaign period (N=7124) to investigate how current events influenced misconception prevalence during this time.

**Results:**

A random forest model best identified misconceptions with an accuracy of 82% from blind validation and found that 37% of the UK tweets (N=7124) about dementia across the campaign period were misconceptions. From this, we could track how the prevalence of misconceptions changed in response to top news stories in the United Kingdom. Misconceptions significantly rose around political topics and were highest (22/28, 79% of the dementia tweets) when there was controversy over the UK government allowing to continue hunting during the COVID-19 pandemic. After our campaign, there was no significant change in the prevalence of misconceptions.

**Conclusions:**

Through codevelopment with carers, we developed an accurate ML model to predict misconceptions in dementia tweets. Our awareness campaign was ineffective, but similar campaigns could be enhanced through ML to respond to current events that affect misconceptions in real time.

## Introduction

### Overview

Negative language and opinions concerning dementia are common on social media platforms [1]. On Twitter, dementia is ridiculed, and stigma surrounding the condition is perpetuated [2]. Stigma toward dementia has been attributed to many different factors, including the loss of independence and functioning the condition can cause [3]. An important factor identified from a systematic review is the myths and misconceptions surrounding dementia; this lack of education around the truth of this condition leads to people forming negative, incorrect beliefs about the condition that are represented by stigma [4]. These misconceptions have also been said to directly influence how communities and families respond to people with dementia [5]. In our past work investigating negative language around dementia on Twitter alongside carers, we found that these misconceptions underlie the negative comments found on the platform and concluded that addressing these directly would help promote awareness and education for dementia rather than simply correcting negative language represented by stigma [6]. With a daily average of 500 million tweets [7], identifying misconceptions quickly can only be carried out with machine learning (ML) [8]. Studies have found that ML models are as accurate as humans in recognizing stigma toward bipolar disorder and general mental health issues in social media posts [9,10] and stigma toward dementia in tweets [2]. However, although Oscar et al [2] attempted to sort tweets into a wide range of categories, we focus solely on the identification of misconceptions, with the full involvement of care partners for those living with dementia to maximize involvement of the community [11,12] and to minimize bias in supervised ML [13]. As supervised models are trained on a given set of classifications, we argue that these classifications should be curated with the community.

Only identifying misconceptions will not change public perceptions; however, by identifying people who are posting them on Twitter, these people can be targeted by an educational awareness campaign. To our knowledge, this form of campaign has not yet been undertaken on a social media platform. Similar campaigns around dementia have either focused on awareness around risks for developing dementia [14] or have been delivered through other mediums [15]. An awareness campaign on social media for dementia would therefore be more comparable with campaigns that reduce stigma and misconceptions of mental health problems such as the *Time to Change *antistigma campaigns [16]. These have been effective at increasing positive attitudes toward mental health and reducing discrimination by 11.5% [17,18]. This has in turn helped those with mental health difficulties feel more able to approach mental health services [19]. Although dementia is a neurodegenerative disease, rather than a mental health condition, these findings suggest that an awareness campaign run on Twitter could be effective in reducing misconceptions around dementia. However, these campaigns are run over long periods, and other factors, such as global events, may also mediate or affect this change (eg, Budenz et al [20] showed that mental health stigma significantly increases after a shooting). We therefore sought to track global events in relation to our campaign to observe how our campaign works in a real-world environment where news stories can shape discussions on social media.

We aimed to codevelop a supervised ML model that can detect dementia misconceptions on Twitter with dementia care partners central to the analytical pipeline and to co-design and then deploy an awareness campaign on Twitter to address these misconceptions. Furthermore, we aimed to use the ML model to evaluate the effectiveness of our campaign in reducing misconceptions and track global events that affected misconceptions during the campaign period.

### Background

This study is built upon our previously published work that qualitatively examined conversations about dementia on Twitter, working with carers of people living with dementia [6]. We held 3 focus groups with them, across which, they defined search terms for finding both negative and neutral tweets about dementia and developed a framework of 6 categories to classify tweets about dementia with 3 misconception categories and 3 neutral categories. A set of 1500 tweets was rated by care partners into these categories, 6 of them, each categorizing 250. Our previous study [6] covered a thematic analysis of the tweets rated as misconception categories by our group of care partners. For this study, we carried forward the obtained search terms to collect further tweets about dementia and used the set of 1500 categorized tweets by carers to develop an ML model based on their choice of categorization.

## Methods

### Design

This was a mixed methods study using participatory methods across 2 stages.

Stage 1 involved developing an ML model to distinguish between misconceptions and neutral tweets; stage 2 involved developing, deploying, and evaluating an awareness campaign to tackle dementia misconceptions.

We sought carer opinions across both stages to ensure our methodology was grounded in their perspective [11,12].

### Ethics Approval

The study was granted ethics approval from the King’s College London Psychiatry, Nursing, and Midwifery Research Ethics Committee (reference HR-19/20-14,565).

### Participants

Participants (care partners for people living with dementia) were recruited if they had unpaid experience of caring for someone close to them with a diagnosis of dementia from the Maudsley Biomedical Research Centre’s dementia research advisory group (MALADY) [21] and Join Dementia Research, a United Kingdom–wide web-based platform hosted by the National Institute of Health Research. Participants were excluded before data collection if they were unable to give consent or were aged <18 years. Participants were asked for their demographic information so we could provide characteristics of the carers codeveloping our model and campaign (Table 1).

**Table 1:** Participant characteristics (N=7).

Characteristics			Participants
Sex (female), n (%)			5 (71)
Age (years), mean (SD)^a^			63.33 (11.79)
**Ethnicity, ** **n (%)**			
	White British	6 (86)	
	Black or Black British	1 (14)	
**Employment status, n (%)^a^**			
	Employed (part time)	1 (14)	
	Self-employed	1 (14)	
	Retired	3 (43)	
	Employment and Support Allowance	1 (14)	
Years being a carer, mean (SD)^a^			8.83 (6.59)

^a^n=1; missing data.

### Tweet Extraction (Data Collection)

In our previous work, we held a focus group with carers to generate a list of dementia-related keywords, both negative and neutral, for tweet extraction [6]. For this focus group, participants first discussed their experiences with dementia being mentioned on Twitter in a 45-minute discourse facilitated by a research assistant. Afterward, participants were each given an iPad that they used to browse Twitter and were told to input search terms they thought might bring up either negative or neutral tweets about dementia. They then examined the tweets that came up from their search and noted how relevant they felt each search term they used was. Participants could freely discuss this task with each other as they completed it. They collectively agreed upon the most useful keywords that brought up both negative and neutral tweets about dementia. These were used to extract tweets using Twitter’s streaming application programming interface [22] with “Tweepy” [23] over 2 extraction phases. The final keywords are indicated in Table S1 in Multimedia Appendix 1 [2,24-29].

The first extraction was performed in our previous study and provided tweets for our carers to rate and were extracted over a 3-day period (February 4, 2020, to February 7, 2020) to ensure that the tweets were not overly affected by a particular daily event. We collected 48,211 tweets relating to dementia: 35,704 (74.06%) using neutral terms and 12,507 (25.94%) using negative terms. A random sample of 750 tweets with negative keywords and 750 with neutral keywords was extracted for the development of our ML models (N=1500).

**Figure 1:**
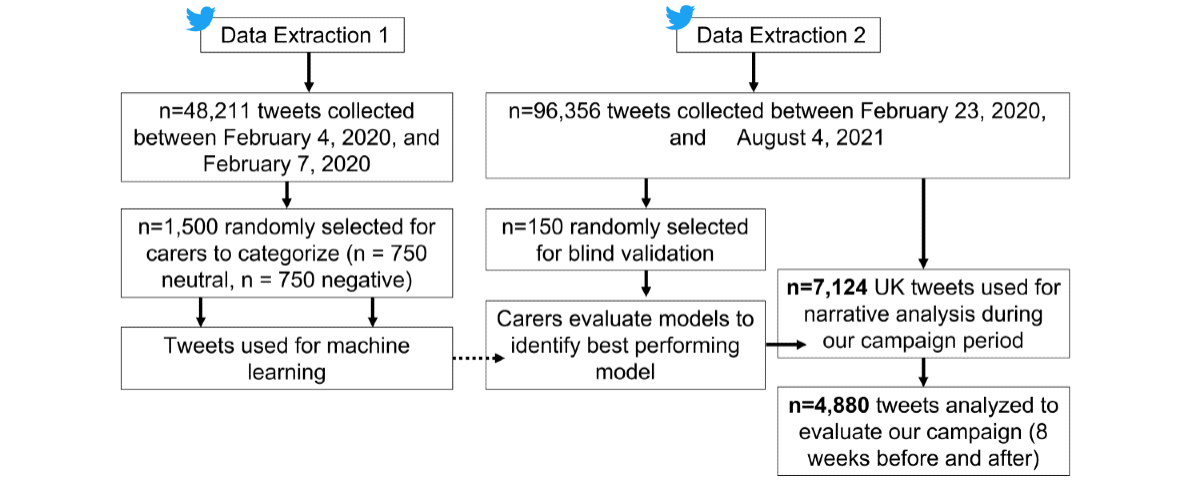
Tweet extraction diagram.

The second extraction consisted of 96,356 tweets using the same keywords. We used subsets of this sample to (1) validate our best performing models, (2) explore differences in features for our best performing model, (3) evaluate the effectiveness of our campaign, and (4) understand how global events affected misconceptions during our campaign. We used a much larger date range for the second extraction (February 23, 2020, to April 8, 2021) to ensure that enough tweets from the United Kingdom were extracted, particularly for data collection before (February 23, 2020, to December 2, 2020) and after (January 28, 2021, to April 8, 2021) our campaign. Figure 1 provides an overview of our data sets and procedure.

All tweets collected were original, not retweets. In line with community principles on ethical data practices guidance, all tweets viewed by participants were anonymized to avoid identifying specific individuals [30]. Anonymization included manually removing screen names; specific individuals being mentioned in tweets were censored, unless they were a particular public figure (eg, Donald Trump). It was decided that a mentioned individual was a “public figure” through looking up their username, and if their account was “verified” (representing the account being both genuine and notable), they were deemed to be a public figure.

### Procedure

#### Stage 1: ML

##### Participatory Involvement

Our model developments had oversight from 7 care partners who attended focus groups and categorized 1500 tweets into neutral and misconception themes. These categories were taken from data used in our previous study [6], where participants built and refined a framework of categories across 2 focus groups, which they then used to rate a set of 250 tweets that they were given (6/7, 86% of the original carers did this process). Half of the categories (3/6, 50%) were agreed upon as different forms of misconceptions and the other half (3/6, 50%) were agreed as different forms of neutral tweets (further detail reported in the study by Hudson and Jansli [6]). We therefore could take each half of the categories and use this set of data as tweets rated as misconceptions, neutral, or neither for the purpose of this study. The number of tweets rated by each participant was collectively decided by the carers as a sample size they could comfortably categorize manually. This size was also manageable from the standpoint of fact-checking tweets, which we left up to the carers’ discretion. Carers identified *“*features*”* (words or characteristics of tweets) that indicated whether a tweet was a misconception or a neutral tweet. They were told about the nature of features in ML, as we did this to ensure that their involvement at this stage was informed, and they fully understood their contribution to the ML model through this task. They were shown a set of tweets rated by another participant and asked to identify any features that they felt indicated whether a tweet was a misconception and how reliable the feature was. The features considered the most reliable were then taken forward by us to be used in the ML model (Table S2 in Multimedia Appendix 1). Carers also evaluated and selected the best model through a blind validation exercise. Finally, they emphasized accuracy and the number of false negatives to be the key parameters for comparing the performance of different models. Through this, we ensured that our models could be held up to the carers’ standards and would therefore be developed according to what they felt was most important.

Importantly, the carers are included as authors on this paper and, as such, have read through and have been able to make comments throughout the writing of this manuscript. Through this, we ensure that we have successfully codeveloped our ML model, awareness campaign, and the paper itself, with the carers.

##### Preprocessing of Tweets

In accordance with the literature, tweets were preprocessed, which involved them being lemmatized first [31]. This ensured the words in the tweets were in their stem form (eg, “depression,” “depressed,” and “depressing,” would all be converted into “depress”); this removed typos and focused on the meaning of words. We then removed “stop words” to reduce noise as done in previous work [31] and tagged each tweet with the appearance of carer-identified features and extracted 10 additional features based on the literature. This included sentiment (positive or negative tone) and subjectivity (factual to subjective) scores via Python’s “TextBlob” library [32]. The other 8 features were tweet descriptive; for example, the length of the tweet [33] and average word length [34] (Multimedia Appendix 1).

Natural language processing methods converted the tweets into their numerical form [35] and we used term frequency–inverse document frequency [24] to vectorize our training set with the default settings in the “Scikit-Learn” library in Python [36]. This generated a data set of features to identify words within the training set that were related to carer-rated misconceptions (Multimedia Appendix 1).

##### Development of the Supervised ML Models

Given the novelty of this work, we compared the ability of 4 classifiers previously used in health data [37] to test their ability to predict misconceptions. The classifiers used were random forest [38], gradient boosted decision tree [39], support vector machine (SVM) with radial basis function, and SVM with linear kernel [40-42]. Each classifier was created with a 5-fold cross-validation. Hyperparameter optimization was performed for each model, prioritizing accuracy and false negatives while also considering recall and precision. For the random forest and gradient boost, the parameters optimized were the maximum depth and the number of estimators. For the SVMs, the cost function was optimized. The algorithms for our ML were trained and tested using Scikit-learn (version 0.24; Python Software Foundation) in the programming language Python (version 3.9.0) [43].

##### ML Blind Validation

As models can perform at similar levels of accuracy during testing [44], we tested levels of agreement between our model and carers by implementing a validation phase for the top 2 models. This serves as further testing for our ML model on a set of tweets independent from those used for training, which is commonly used in validating ML models [45,46]. Our past research has shown that this additional step can help clarify a small difference in accuracies and demonstrate a clearer difference in performance between top-performing models, confirming that this is an important step [31]. We randomly selected 150 tweets from our second sample of 96,356 tweets and split them into 3 batches of 50. A total of 5 care partners then categorized these tweets as misconceptions or neutral tweets. Carers were not shown the model’s predicted category (ie, blind validation). When 2 carers agreed on a tweet’s category, we took this as the final agreed classification. Tweets without an agreement on category were rated by another carer who decided the final classification. Final carer classifications were compared with our model classifications to investigate agreement.

#### Stage 2: Campaign to Reduce Misconceptions on Twitter

##### Participatory Involvement

Carers codeveloped a campaign to combat misconceptions. This was done in two stages:

1. Participants were shown previous dementia awareness campaigns and reflected on what was good and bad about each of them. They then suggested several different focus areas for a campaign, detailing what it should include and how it would address the issue of misconceptions.
2. We combined the suggested focus areas for a campaign, looking for overlaps between suggestions and, from this, developed 3 campaign concepts, focusing on the way language around dementia needs to change, dispelling specific myths or telling the stories of people behind the diagnosis. Each carer assessed the campaign concepts and made suggestions about them including specific quotes to use. We then created infographics for the campaign concept that most carers thought was the best, incorporating selected quotes that were suggested.

##### Campaign Deployment

We deployed our campaign infographics via our Twitter account “@DementiaReality” for a period of 8 weeks, from December 3, 2020, to January 27, 2021. The campaign targeted UK-based individuals who had previously posted tweets with negative dementia keywords (Table S1 in Multimedia Appendix 1). Our campaign was followed by a poll which asked, “How has a recent dementia tweet made you think differently about dementia?” and Twitter users responded through four choices (“more positively,” “more negatively,” “no difference,” or “didn’t see it”). We opted to ask about “any dementia tweet” to ensure that we did not prompt them to remember the original tweet.

##### Campaign Evaluation

We evaluated our campaign by applying our carer validated ML model through UK-based tweets, posted from 8 weeks before to 8 weeks after our campaign, from our second tweet data set (7124 of 96,356 tweets) to compare the prevalence of dementia misconceptions on Twitter before and after our campaign. Tweets were identified as being from the United Kingdom through the use of geographic longitude and latitude co-ordinates of a reference point (an address specified for all people living in a particular area) associated with the user who posted the tweet.

### Data Analysis

#### Stage 1

##### Manual Coding of Tweets

We performed independent sample 2-tailed *t* tests and chi-square tests to investigate which features (both carer-identified and literature-defined features) significantly differed between misconceptions and neutral tweets in 1414 tweets. In addition, we also made use of the sklearn library’s feature selection with family-wise error in Python to compare this algorithm with our manual tests and confirm their validity. Only statistically significant features were used in our ML model to improve accuracy and reduce noise [47].

##### Evaluation of the ML Model

We evaluated our models based on accuracy and false negatives and standard ML metrics [48]. Accuracy answers the question, *“*Overall, how often is the model correct?*”* and the number of false negatives highlights cases where the model incorrectly classified a tweet as neutral.

##### ML Blind Validation

To assess the levels of agreement between carers and our 2 best ML models, we performed cross-tabulations, calculated a Cohen κ statistic and a 2×2 chi-square to assess the difference between the models’ accuracies by examining the proportion of correct ratings.

#### Stage 2

We investigated the effect of our campaign on (1) the prevalence of misconceptions among UK Twitter users who discuss dementia and (2) sentiment. We tested whether these outcomes differed in UK-based tweets 8 weeks before and after the campaign, using chi-square tests or 2-tailed *t* tests where appropriate.

Twitter does not allow us to view the users who have been shown our campaign, so it is not possible to directly assess the level of misconceptions of those who had been shown the campaign. To address this, we examined the frequency of misconceptions tweeted by a given user within our second set of extracted tweets. To do this, we classified tweets in this data set using our ML model and examined all identified misconceptions. We separated the tweets by username and identified the average number of days between each user’s misconception tweets. This way, we could demonstrate whether people consistently tweet misconceptions and, therefore, that they would likely to be targeted by the campaign and appear in our evaluation.

We computed the rolling 3-day average of the prevalence of misconceptions and sentiment to investigate changes over our 6-month study period (8 weeks before, 8 weeks during, and 8 weeks after the campaign) and used a time series trend to identify any external influences [49]. To understand how current affairs affected misconceptions, we calculated the mean and SD for sentiment and prevalence at each day across this 6-month period and investigated time points where sentiment and prevalence were –2 to +2 SD from the mean; that is, statistical outliers at 95% probability [50].

## Results

### Stage 1

#### Feature Extraction

Carers identified 18 features, 13 (72%) for misconceptions and 5 (28%) for neutral tweets. Carers associated the words “demented” and “senile” as belonging to misconception tweets, as well as tweets where “Donald Trump” and “Nancy Pelosi” are mentioned. Tweets with a URL or those with the words “research” or “memory” were associated with neutral tweets.

#### Feature Analysis

##### Carer-Identified Features

Of the 18 features carers identified, 9 (50%) significantly distinguished misconceptions from neutral tweets. These included the mention of Donald Trump (11.97% in misconceptions vs 0% in neutral tweets; *χ*^2^_1_=81.6; *P*<.001) and the occurrence of the word “demented” (46.98% in misconceptions vs 0.16% in neutral tweets; *χ*^2^_1_=399.9; *P*<.001).

##### Literature-Identified Features

We found significant differences in 8 of the 10 (80%) features with misconceptions being more negative in sentiment (mean −0.04, SD 0.30 vs mean 0.16, SD 0.28; *t*_1,412_=12.94; *P*<.001) and shorter (mean characters 139.31, SD 73.12) than neutral tweets (mean 178.97, SD 63.04; *t*_1,412_=13.71; *P*<.001).

A full list of features and their significance tests are provided in Tables S3 and S4 in Multimedia Appendix 1. The significance of features from our test run by the “sklearn” algorithm was compared with the manual tests in Table S5 in Multimedia Appendix 1; the difference was minimal, so we proceeded with our manual tests in mind.

#### Manual Coding

Of the 1500 tweets presented to carers, 86 (5.73%) could not be categorized because the carers felt they could not be sure whether the tweet was a misconception, leaving 1414 for ML: 637 (45.04%) neutral and 777 (54.95%) misconceptions.

#### ML Model Evaluation

We evaluated our ML models based on various parameters (Table 2). The SVM with a linear kernel and the random forest performed equally well in terms of accuracy (96% each), but the random forest had 7 false negatives, which was slightly less than the SVM with a linear kernel which had 10. Hyperparameter optimization led to our SVM with linear kernel having a cost function of 0.1 and our random forest having a maximum depth of 25 and 500 estimators.

**Table 2:** Machine learning model comparison.

Parameter	RF^a^	GB^b^	SVM^c^: RBF^d^	SVM linear
Accuracy^e^	0.96	0.95	0.96	0.96
Misclassification rate	0.04	0.05	0.04	0.04
Sensitivity	0.96	0.94	0.93	0.94
Specificity	0.97	0.96	0.99	0.99
False positive rate	0.03	0.04	0.01	0.01
Precision	0.97	0.97	0.99	0.99
False negative rate	0.04	0.06	0.07	0.06
False negatives^e^	7	10	11	10
False positives	3	5	2	1
Area under the receiver operating characteristic curve	0.97	0.95	0.96	0.96

^a^RF: random forest.

^b^GB: gradient boost.

^c^SVM: support vector machine.

^d^RBF: radial basis function.

^e^These parameters were the primary ones used for assessing model performance.

#### ML Blind Validation

Carers considered 72% (108/150) of the tweets to be misconceptions in the validation data set. We then applied our top two models (SVM with a linear kernel and random forest) to these 150 tweets to select the best performing model:

- SVM with a linear kernel and carers: there was moderate agreement (Cohen κ= 0.538, 95% CI 0.403-0.673; *P*<.001) with agreement on 79% of ratings; there were 26 false negatives. The SVM predicted that 58.6% (88/150) of the tweets were misconceptions.
- Random forest and carers: there was a moderate agreement (Cohen κ=0.581, 95% CI 0.442-0.720; *P*<.001), with agreement on 82% of the ratings and 18 false negatives. The random forest predicted that 72% (108/150) of the tweets were misconceptions.

The random forest was significantly more accurate than the SVM with a linear kernel (n=150; *χ*^2^_1_=79.9; *P*<.001).

### Stage 2

#### Campaign Deployment

**Figure 2:**
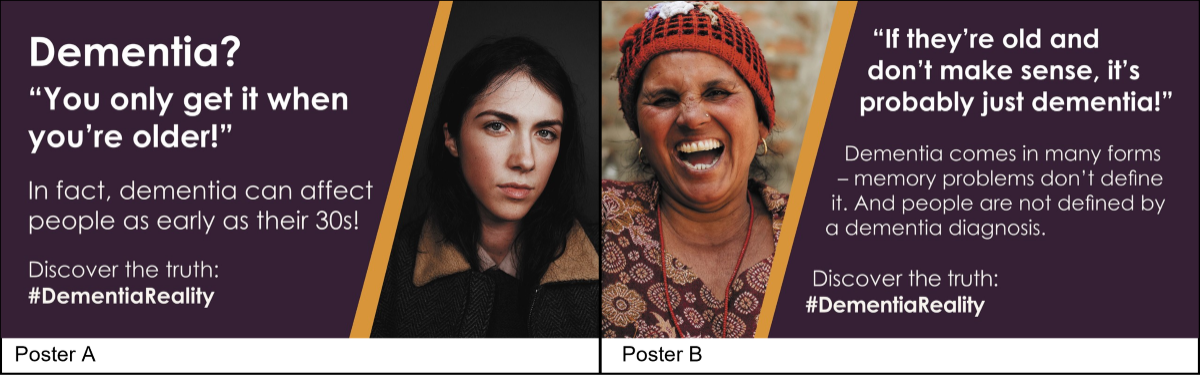
Our campaign posters advertised on Twitter.

Our campaign addressed common dementia misconceptions and outlined facts (Figure 2). The graphics were implemented by a graphic designer with quotes suggested by the carers and had a link to more information about dementia on the Alzheimer’s Research UK website.

These campaign posters were delivered to 239,360 UK Twitter users who saw at least one of them, and 2.12% (5071/239,360) of the users responded to our evaluation question. Furthermore, 8.05% (408/5071) of the users reported that the campaign had a positive impact, 5.70% (289/5071) reported a negative impact, 10.89% (552/5071) reported no impact, and most (3822/5071, 75.37%) users did not remember seeing it.

#### Campaign Evaluation

We classified UK tweets spanning 8 weeks before the start of our campaign and 8 weeks after the end of our campaign (a total of 16 weeks, N=4880 tweets; Table 3). A chi-square test of independence between the 8-week periods before and after our campaign found no significant difference in prevalence of misconceptions (N=4880; *χ*^2^_1_=0.8; *P*=.36). There was also no statistically significant difference in sentiment before and after the campaign *(t*_4878_=1.219; *P*=.22).

**Table 3:** Differences in outcome measures 8 weeks before and after our campaign.

Period	Before the campaign (October 8, 2020, to December 2, 2020)	After the campaign (January 28, 2021, to March 24, 2021)
Total number of tweets (n)	2877	2003
Number of misconceptions, n (%)	1035 (35.97)	746 (37.24)
Sentiment, mean (SD)	0.09 (0.30)	0.08 (0.30)

#### UK-Based Dementia Tweets

We found the prevalence of misconceptions in our set of 7124 UK tweets to be 37%.

##### Carer-Identified Features

The word “senile” appeared in 13.6% of the misconceptions compared with 0% in neutral tweets (N=7124; *χ*^2^_1_=640.5; *P*<.001). Tweets with the appearance of the word “caregiver” did not significantly differ between misconceptions and neutral tweets (N=7124; *χ*^2^_1_=3.6; *P*=.06).

##### Literature-Defined Features

Sentiment was significantly higher in neutral tweets (mean 0.14, SD 0.29) compared with misconceptions (mean −0.03, SD 0.28; *t*_7,122_=5.72; *P*<.001) and word count was significantly shorter in misconceptions (mean 21.90, SD 14.50) compared with neutral tweets (mean 33.33, SD 12.85; *t*_7,122_=33.56; *P*<.001).

A full list of feature significances is provided in Tables S6 and S7 in Multimedia Appendix 1.

#### Frequency of Misconceptions

Our model identified 45,865 tweets as misconceptions within our second set of 96,356 tweets. Table 4 details how often users usually tweeted about misconceptions.

The vast majority of users (45,011/45,865, 98.14%) only tweeted misconceptions as a one-off event, multiple times within a day, or at most within a month. Most users did not continue to tweet misconceptions long after they had first done so.

**Table 4:** The frequency of users posting misconceptions.

Frequency	Tweets, n (%)
One-off misconception tweet	39,837 (86.85)
Multiple within 1 day	757 (1.65)
Within 1 month	4417 (9.63)
Over a month	854 (1.86)

#### How Current Affairs Affected Misconceptions and Sentiment Across Our Campaign Period

**Figure 3:**
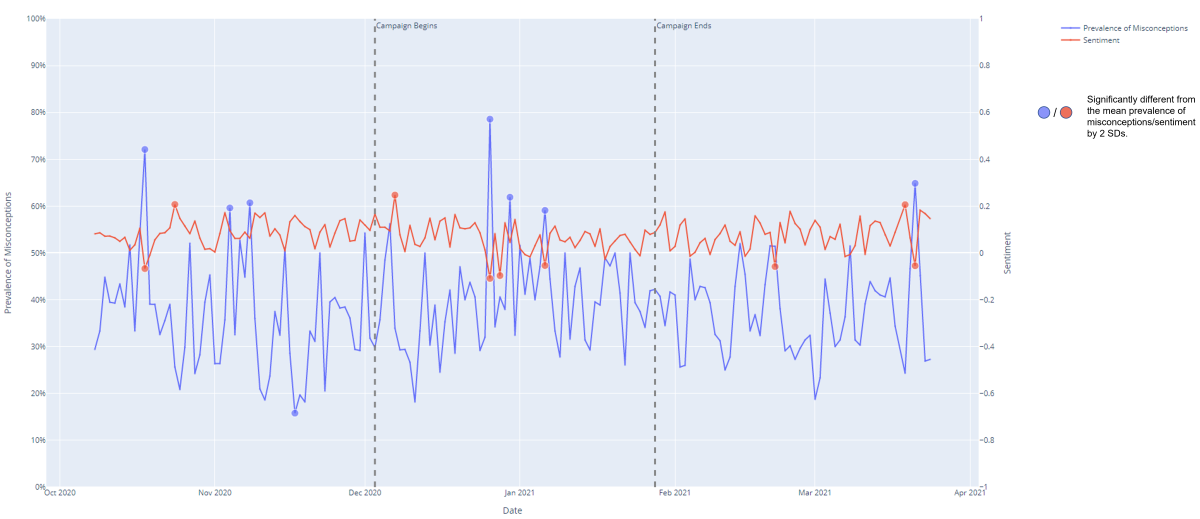
Graph of the prevalence of misconceptions and average sentiment on each day for 8 weeks before, during and after our campaign.

We identified dates where prevalence was 2 SDs above or below the mean daily prevalence of misconceptions (mean 38%, SD 11%); that is, <17% or >59%. In total, 8 time points fulfilled these criteria; 7 above and 1 below. We also identified dates where sentiment was 2 SDs away from the mean (0.08, SD 0.06); that is, <−0.04 or >0.20. We identified 9 time points: 3 above and 6 below. These points are indicated on Figure 3.

Misconceptions in UK tweets were high, and sentiment was low on the day former president Donald Trump announced that he would rather leave the United States than admit defeat to President Biden [51] (October 18, 2020), with 72% of the tweets being misconceptions and the average sentiment being −0.07. Misconceptions were also high and sentiment low during COVID-19–related events. These included the controversy over the COVID-19 pandemic restriction exemption for hunting in the United Kingdom [51] (December 26, 2020), with 79% of the tweets containing misconceptions, the average sentiment being −0.11 and when reports of a nurse breaking down over empty supermarket shelves went viral [51] (March 21, 2021), with 64% of the tweets containing misconceptions, the average sentiment being −0.05.

## Discussion

### Principal Findings

We codeveloped and tested an ML model to automatically classify dementia misconceptions with 96% accuracy and a campaign to dispel dementia myths and educate Twitter users on stigmatizing language. We ensured that carers were at the core of our analyses through participatory methods throughout the study. We also show how misconceptions peak and trough as global events shape the Twitter conversations.

Training a model from carer opinions and involving them throughout the study (also including them as authors) has yielded a unique perspective on misconceptions about dementia and how they impact those affected by dementia. This approach differs from previous ML approaches that only used researcher-defined themes [2]. Many features in our study are well established as stigmatizing words or phrases in the literature, such as calling those with the condition “demented,” “senile,” or diminishing them as “not being all there” [52,53]. However, we also show that an indicator of misconceptions was weaponizing the disease to insult older public figures, most notably politicians, such as Donald Trump; this is in line with the findings of our previous study [6].

Our work improves on previous modeling that detected ridicule as a form of negativity in dementia tweets at 90% accuracy [2]. This demonstrates the value in using larger data sets for training models (eg, Oscar et al [2] used only 331 tweets), as larger training data sets expose a model to a heterogeneous range of language. We also deployed a validation stage that is not commonly used, as noted in a systematic review by Wongkoblap et al [54], so this extra step has no context of comparison within the literature. Our model performed well and was firmly established in the opinions of carers with 96% accuracy, highlighting the effectiveness of community involvement in the ML pipeline.

The campaign we developed did not yield similar benefits from carer involvement and showed clear signs of not being effective. From just our initial polls, we could see the campaign had not left much of an impact, with the vast majority of people not remembering seeing it. Similar campaigns on social media usually assess general awareness of campaigns, without knowing whether that person has seen it before, and so this lack of awareness is uniquely poor [14]. This may be because of the nature of advertisements on Twitter, which are a natural part of a person’s feed and thus can easily be scrolled past. In combination with the fact that our funding only allowed for our campaign to be shown once to most people, our campaign was likely not able to have much impact. As such, our finding of little reduction in the prevalence of misconceptions is not surprising, showing that our campaign was ineffective.

Our finding that levels of misconceptions change in response to news events also shows how external factors should ideally be taken into account when running a campaign. By using ML to categorize large amounts of tweets in a short time, notable changes can be tracked, and the news stories associated can be identified, allowing for real-time responses in the campaign, potentially enhancing its effectiveness.

### Strengths and Limitations

This study is built on the firm foundation and involvement of those caring for people living with dementia. The opinions of carers were used to fully develop our ML model and our campaign. This perspective is key to classifying dementia misconceptions, as carers are greatly affected by them, and so can provide a unique perspective in identifying tweets that would be the most harmful. None of our participants had a diagnosis of dementia, and this would be an important perspective to incorporate into future work where appropriate. In addition, ML models such as the ones used in this study benefit from larger training sets; given the number of carers and tweets that could reasonably be rated, it is possible that our sample resulted in overfitting. Future research should incorporate larger samples. It is difficult to fully account for spelling mistakes and their frequency within tweets. Although lemmatization accounts for a great deal of these, some spelling mistakes would make it more difficult for our model to correctly use these words. Furthermore, in future research, different approaches to preprocessing and lemmatization could be used, such as the Python library Bidirectional Encoder Representations from Transformers, which has specific uses appropriate for tweets.

Twitter campaigns must competitively bid for “ad space” to show advertisements to users. This may mean that the target audience only has a campaign advertisement appear approximately once on their feed and may explain why 75% of the users did not remember seeing our campaign. Twitter does not provide the names of those targeted by our campaign, so we could not examine the tweets of specific people. Despite this, by examining the general discourse around dementia from tweets posted by people in the United Kingdom, we could indirectly assess how our campaign affected the prevalence of misconceptions: this indirect assessment of the audience being a usual way of assessing web-based campaign effectiveness [14-16]. As we found that the vast majority of users did not continue to tweet misconceptions, long after they had first done so, our study is limited by its inability to directly assess those who viewed our campaign. However, our method of extraction did not provide an exhaustive list of tweets from each user, and as such, this does not necessarily assess all tweets of every user; it is therefore possible that users did indeed tweet misconceptions over time. In the future, it would be important to consider directly assessing users and ensuring that they tweet misconceptions over a long period. Future work must also ensure a competitive campaign budget so that advertisements are shown to users multiple times, as sheer repetition may then have an effect. It is not possible to distinguish world events from the effect of our campaign through this study. Our examination of news stories suggests that they can have an impact on the use of language related to dementia in discussions on Twitter.

### Conclusions

This study showed how accurate ML models can be developed alongside carers of people with dementia, highlighting the effectiveness of codevelopment alongside individuals with relevant personal experience. Unfortunately, our campaign seemed unimpactful and ineffective in practice, but from this, we can see the potential in using ML models to assess campaigns. Such assessment could be done in real time, combined with tracking news stories that affect levels of misconceptions, which could be used to tailor the campaign to relative news stories.

## Appendix

Multimedia Appendix 1 Details about feature extraction and preprocessing of tweets, as well as detailed results about significant features.

## Abbreviations

ML: machine learning
NIHR: National Institute for Health Research
SVM: support vector machine

## Data Availability

Data are available upon request from the corresponding author. The scripts used are available on GitHub [55].

## References

[1] Robinson P, Turk D, Jilka S, Cella M (2018). Measuring attitudes towards mental health using social media: investigating stigma and trivialisation. Soc Psychiatry Psychiatr Epidemiol.

[2] Oscar N, Fox PA, Croucher R, Wernick R, Keune J, Hooker K (2017). Machine learning, sentiment analysis, and tweets: an examination of Alzheimer's disease stigma on Twitter. J Gerontol B Psychol Sci Soc Sci.

[3] Cipriani G, Borin G (2015). Understanding dementia in the sociocultural context: a review. Int J Soc Psychiatry.

[4] Cahill S, Pierce M, Werner P, Darley A, Bobersky A (2015). A systematic review of the public's knowledge and understanding of Alzheimer's disease and dementia. Alzheimer Dis Assoc Disord.

[5] Morgan DG, Semchuk KM, Stewart NJ, D’Arcy C (2002). Rural families caring for a relative with dementia: barriers to use of formal services. Social Sci Med.

[6] Hudson G, Jansli SM, Erturk S, Morris D, Odoi CM, Clayton-Turner A, Bray V, Yourston G, Clouden D, Proudfoot D, Cornwall A, Waldron C, Wykes T, Jilka S (2022). Investigation of carers' perspectives of dementia misconceptions on Twitter: focus group study. JMIR Aging.

[7] Twitter usage statistics. Internet Live Stats.

[8] Li A, Jiao D, Zhu T (2018). Detecting depression stigma on social media: a linguistic analysis. J Affect Disord.

[9] Tokmic F, Hadzikadic M, Cook JR, Tcheremissine OV (2018). Development of a behavioral health stigma measure and application of machine learning for classification. Innov Clin Neurosci.

[10] Budenz A, Klassen A, Purtle J, Yom Tov E, Yudell M, Massey P (2020). Mental illness and bipolar disorder on Twitter: implications for stigma and social support. J Ment Health.

[11] Wykes T (2014). Great expectations for participatory research: what have we achieved in the last ten years?. World Psychiatry.

[12] Rose D (2018). Participatory research: real or imagined. Soc Psychiatry Psychiatr Epidemiol.

[13] (2017). Machine Learning: the Power and Promise of Computers That Learn by Example.

[14] Van Asbroeck S, van Boxtel MP, Steyaert J, Köhler S, Heger I, de Vugt M, Verhey F, Deckers K (2021). Increasing knowledge on dementia risk reduction in the general population: results of a public awareness campaign. Prev Med.

[15] Phillipson L, Hall D, Cridland E, Fleming R, Brennan-Horley C, Guggisberg N, Frost D, Hasan H (2019). Involvement of people with dementia in raising awareness and changing attitudes in a dementia friendly community pilot project. Dementia (London).

[16] Evans-Lacko S, Corker E, Williams P, Henderson C, Thornicroft G (2014). Effect of the Time to Change anti-stigma campaign on trends in mental-illness-related public stigma among the English population in 2003-13: an analysis of survey data. Lancet Psychiatry.

[17] Henderson C, Thornicroft G (2009). Stigma and discrimination in mental illness: time to change. Lancet.

[18] Sampogna G, Bakolis I, Evans-Lacko S, Robinson E, Thornicroft G, Henderson C (2017). The impact of social marketing campaigns on reducing mental health stigma: results from the 2009-2014 Time to Change programme. Eur Psychiatry.

[19] Corker E, Hamilton S, Henderson C, Weeks C, Pinfold V, Rose D, Williams P, Flach C, Gill V, Lewis-Holmes E, Thornicroft G (2013). Experiences of discrimination among people using mental health services in England 2008-2011. Br J Psychiatry Suppl.

[20] Budenz A, Purtle J, Klassen A, Yom-Tov E, Yudell M, Massey P (2019). The case of a mass shooting and violence-related mental illness stigma on Twitter. Stigma Health.

[21] Support for Researchers. NIHR Maudsley Biomedical Research Centre.

[22] Docs. Developer Platform.

[23] API. Tweepy.

[24] Salton G, McGill MJ (1983). Introduction to Modem Information Retrieval.

[25] Delahanty RJ, Alvarez J, Flynn LM, Sherwin RL, Jones SS (2019). Development and Evaluation of a Machine Learning Model for the Early Identification of Patients at Risk for Sepsis. Ann Emerg Med.

[26] Neppalli VK, Medeiros M, Caragea C, Caragea D, Tapia A, Halse S (2016). Retweetability analysis and prediction during Hurricane Sandy. Proceedings of the ISCRAM 2016 Conference.

[27] Gretry A, Davis SW, Horvath C, Belei N (2017). How tweet readability and brand hedonism affect consumer engagement. Advances Consum Res.

[28] Farías Dih, Patti V, Rosso P (2016). Irony Detection in Twitter. ACM Trans Internet Technol.

[29] Giving you more characters to express yourself. Twitter.

[30] Green L (2018). Community principles on ethical data sharing presented at Bloomberg's data for good exchange. DOMINO.

[31] Jilka S, Odoi CM, van Bilsen J, Morris D, Erturk S, Cummins N, Cella M, Wykes T (2022). Identifying schizophrenia stigma on Twitter: a proof of principle model using service user supervised machine learning. Schizophrenia (Heidelb).

[32] Du J, Xu J, Song H, Liu X, Tao C (2017). Optimization on machine learning based approaches for sentiment analysis on HPV vaccines related tweets. J Biomed Semantics.

[33] Gulliver A, Griffiths KM, Christensen H, Mackinnon A, Calear AL, Parsons A, Bennett K, Batterham PJ, Stanimirovic R (2012). Internet-based interventions to promote mental health help-seeking in elite athletes: an exploratory randomized controlled trial. J Med Internet Res.

[34] Crawford M, Khoshgoftaar TM, Prusa JD, Richter AN, Al Najada H (2015). Survey of review spam detection using machine learning techniques. J Big Data.

[35] James G, Witten D, Hastie T, Tibshirani R (2013). An Introduction to Statistical Learning with Applications in R.

[36] Abraham A, Pedregosa F, Eickenberg M, Gervais P, Mueller A, Kossaifi J, Gramfort A, Thirion B, Varoquaux G (2014). Machine learning for neuroimaging with scikit-learn. Front Neuroinform.

[37] Anjaria M, Guddeti R (2014). Influence factor based opinion mining of Twitter data using supervised learning. Proceedings of the 2014 Sixth International Conference on Communication Systems and Networks (COMSNETS).

[38] Breiman L (2001). Random forests. Mach Learn.

[39] Friedman JH (2001). Greedy function approximation: a gradient boosting machine. Ann Statist.

[40] Vapnik V (1995). The Nature of Statistical Learning Theory.

[41] Vapnik V (1998). Statistical Learning Theory.

[42] Cortes C, Vapnik V (1995). Support-vector networks. Mach Learn.

[43] Python homepage. Python.

[44] Wong NC, Lam C, Patterson L, Shayegan B (2019). Use of machine learning to predict early biochemical recurrence after robot-assisted prostatectomy. BJU Int.

[45] Ginart A, Das S, Harris J, Wong R, Yan H, Krauss M, Cavazos-Rehg PA (2016). Drugs or dancing? Using real-time machine learning to classify streamed “dabbing” homograph Tweets. Proceedings of the 2016 IEEE International Conference on Healthcare Informatics (ICHI).

[46] Shah T (2017). About train, validation and test sets in machine learning. Towards Data Science.

[47] Paul S (2020). Python feature selection tutorial: a beginner's guide. DataCamp.

[48] Thieme A, Belgrave D, Doherty G (2020). Machine learning in mental health. ACM Trans Comput Human Interact.

[49] Bryhn AC, Dimberg PH (2011). An operational definition of a statistically meaningful trend. PLoS One.

[50] Dunn P (2021). Scientific Research and Methodology: An Introduction to Quantitative Research in Science and Health.

[51] Wincalendar homepage. Wincalendar.

[52] Milne A (2010). The 'D' word: reflections on the relationship between stigma, discrimination and dementia. J Ment Health.

[53] Swaffer K (2014). Dementia: stigma, language, and dementia-friendly. Dementia (London).

[54] Wongkoblap A, Vadillo MA, Curcin V (2017). Researching mental health disorders in the era of social media: systematic review. J Med Internet Res.

[55] Zenedo.

