# Supplementary material for: Codeveloping and Evaluating a Campaign to Reduce Dementia Misconceptions on Twitter: Machine Learning Study
Source: JMIR Infodemiology. 2022 Nov 22;2(2):e36871. doi: 10.2196/36871 (PMC9987190; doi:10.2196/36871)
Supplement: Multimedia Appendix 1 [file infodemiology_v2i2e36871_app1.docx]

# Multimedia Appendix 1

# Supplementary Material

## 1. Methods

### 1.1 Machine Learning Model Performance

In order to assess the performance of our different models, we calculated a variety of different measures. These were all calculated using four different variables:

True positives (TP): The number of negative tweets that the model correctly identified.

True negatives: (TN): The number of neutral tweets that the model correctly identified.

False positives (FP): The number of neutral tweets the model classified as negative.

False negatives (FN): The number of negative tweets the model classified as neutral.

The measures we calculated were as follows:

Accuracy = The total of correctly classified tweets (TP + TN) divided by the total number of tweets (TP + TN + FP + FN)

Misclassification Rate = The total number of incorrectly classified tweets (FP + FN) divided by the total number of tweets (TP + TN + FP + FN)

Sensitivity = The number of times the model correctly identified a negative tweet (TP) divided by the total number of negative tweets (TP+FN)

Specificity = The number of times the model correctly identified a neutral tweet (TN) divided by the total number of neutral tweets (TN+FP)

False Positive Rate = The number of times the model incorrectly identified a negative tweet (FP) divided by the total number of neutral tweets (FP+TN)

Precision = The number of times the model was correct to classify a tweet as negative (TP) divided by the total number of tweets it classified as negative (TP + FN)

False Negative Rate = The number of times the model incorrectly identified a neutral tweet (FN) divided by the total number of negative tweets (FN+TP)

Area Under Curve (AUC) for Receiver Operating Curve (ROC) = The ROC is a curve that plots the Precision against the False Positive Rate at different threshold values. The AUC is the area under this curve and serves as a measure of how well the model can distinguish between the two categories.

### 1.2 Researcher Feature Engineering

Feature engineering is the application of domain knowledge to potential model inputs, with the goal of creating a feature set that is optimized to predict stigma [1]. Features that were identified by our service user advisory group are indicated with a *. We extracted the following features:

1. *Sentiment:*

The language of each tweet was analysed for its sentiment using a python library called TextBlob. By analysing the language used in the text, sentiment analysis identifies how positive, neutral or negative a text is. Words with a stronger degree of sentiment will have a greater influence on the score and we considered this to be an important feature in stigma detection.

1. *Subjectivity:*

Each tweet was analysed to score the subjectivity of its content again using TextBlob. Sentences that refer to personal opinion, emotion or judgment score closer to 1, whereas objective sentences referring to factual information score closer to 0. We wanted to investigate whether stigmatising tweets were more subjective in nature than non-stigmatising tweets.

1. *Length of tweet:*

Shorter tweets are also associated with stronger opinions [2], and we test whether, stigmatising tweets, which are based more in opinion than fact, are shorter than non-stigmatising tweets.

1. *Punctuation*:*

Each tweet was analysed for the proportion of punctuation it contained to investigate whether stigmatising tweets contained more punctuations (e.g. exclamations marks) than non-stigmatising tweets. The improper use of grammar has been associated with stigma in tweets [3], so we tested suggested how the proportion of punctuation in tweets is associated with stigma.

1. *The number of uppercase words*:*

The YPMHAG noted that anger or rage is quite often expressed by writing in uppercase and we investigated whether stigmatising tweets were characterised by this feature.

1. *The average word length of a tweet:*

This was computed by taking the sum of the length of all the words in a tweet and dividing it by the total length of the tweet. The average word length is an indicator of readability [4] and we test whether stigmatising tweets are more or less readable based on their average word length

1. *The number of words in a tweet:*

Using fewer words is associated with a ‘clear communication goal’ which indicates negative emotion rather than objectivity [5] We tested if stigmatising tweets used fewer words than non-stigmatising tweets.

1. *The number of characters in a tweet:*

Twitter had increased its character limit to prevent people from ‘cramming their thoughts’ [6] It is therefore thought that as a user approaches the character limit (an increase in number of characters), they are having to put more thought into what they say, to ensure it is captured within the limit. We test whether stigmatising tweets are not well thought out as they may contain fewer characters.

1. *The number of hashtags in a tweet**

YPMHAG members indicated that they would come across negative content while using hashtag search options.

1. *The number of numeric characters in a tweet**

Members of the YPMHAG hypothesised that stigmatising content may contain words where a letter is replaced by its numerical form (e.g. the letter ‘A’ replaced with the number ‘4’).

### 1.3 Vectorisation

Each tweet was vectorized using Term Frequency – Inverse Document Frequency (TF-IDF) [7]. This method of vectorizing is based on the weighting of words within the tweet, with the importance of the word within a tweet encapsulated by its weighting. This is considered along with the frequency of the word across all the tweets, where rarer words are given higher values (see equation below).

This weighting encapsulates the amount of information inherent in a word, based on a linguistic observation. For example, a noun or a verb may represent greater meaning but occur less often when compared to function words. In this variant, a weighting based on the document frequency (i.e. the number of tweets containing the word) is multiplied by the frequency of the word in the tweet, as outlined by equation 1.


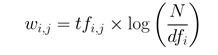


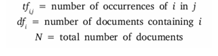


*Equation* *1. The formula to calculate the Term Frequency – Inverse Document Frequency of a text dataset.*

The aim of this was to create feature vectors, where the machine learning algorithm learns to correlate the frequency of certain features (words) in a tweet with the misconception classification ratings given to a tweet. In this way, the supervised machine learning model is used to create the automatic classifier, which is learning to predict misconception classification of a new tweet based on the human coded data it is trained on. The resulting classifier is then used to assign class labels to testing instances (i.e. new tweets) where values of the predictor features are known (i.e. the new tweet itself) but the value of the class label is unknown (i.e. the misconception level of the unrated tweet).

## 2. Results

### 2.1 Search Terms and Features

| **Supplementary Table 1. List of search terms suggested by carers to find negative and neutral tweets** | |
| --- | --- |
| **For Neutral Tweets** | **For Negative Tweets*** |
| *“Dementia”* | *“Dementing illness”* |
| *“Alzheimer’s”* | *“Demented”* |
| *“Alzheimer’s disease”* | *“Senile”* |
| *“Mixed dementia”* | *“Senility”* |
| *“Vascular dementia”* | *“Pre-senile”* |
| *“Lewy body dementia”* | *“Dementia/Alzheimer’s senile/senility/pre-senile”* |
| *“Fronto-temporal dementia”* | *“Dementia/Alzheimer’s sufferer”* |
| *“Dementia/Alzheimer’s memory loss”* | *“Suffering/suffer from Dementia/Alzheimer’s”* |
| *“Dementia/Alzheimer’s cognitive decline”* | *“Dementia/Alzheimer’s hopeless”* |
| *“Young/Early onset dementia/Alzheimer’s”* | *“Dementia/Alzheimer’s affliction/afflicted”* |
| *“Dementia patient”* | *“Dementia/Alzheimer’s tragic”* |
| *“Dementia care”* | *“Dementia/Alzheimer’s burden”* |
| *“Dementia/Alzheimer’s challenging”* | *“Dementia/Alzheimer’s victim”* |
| *“Dementia/Alzheimer’s challenging behaviours”* | *“Dementia/Alzheimer’s doolally”* |
| *Dementia service user* | *“Dementia/Alzheimer’s vulnerable”* |
| *#sharetheorange* | *“Dementia/Alzheimer’s vacant”* |
|  | *“Dementia/Alzheimer’s empty shell”* |
|  | *“Dementia/Alzheimer’s difficult behaviours”* |
| **These terms were also used as keywords to identify the audience of our campaign* | |

| **Supplementary Table 2. List of features carers suggested would indicate a negative or neutral dementia tweet** | |
| --- | --- |
| *Negative* | *Neutral* |
| The word ‘senile’ is in | The word ‘memory’ is in |
| The word ‘demented’ is in | The word ‘café’ is in |
| Donald Trump is mentioned | The word ‘research’ is in |
| Nancy Pelosi is mentioned | A weblink is in |
| The word ‘deranged’ is in | The word ‘caregiver’ is in |
| The word ‘insane’ is in |  |
| The word ‘imbecile’ is in |  |
| The word ‘loon’ is in |  |
| The word ‘crazy’ is in |  |
| The word ‘looney bin’ is in |  |
| The word ‘lunatic’ is in |  |
| The word ‘unhinged’ is in |  |
| The word ‘senility’ is in |  |

### 2.2 Manual Coding

| **Supplementary table 3. Significant differences between neutral and misconception tweets for literature-defined features (Manually coded set (N=1,414)** | | | | | | |
| --- | --- | --- | --- | --- | --- | --- |
|  | Neutral | | Negative | |  |  |
| Feature | Mean | Standard Deviation | Mean | Standard Deviation | *t* | *p* |
| **Sentiment** | **0.16** | **0.28** | **-0.04** | **0.30** | **12.94** | **0.00** |
| Subjectivity | 0.41 | 0.29 | 0.43 | 0.31 | 1.55 | 0.12 |
| **Body Length** | **178.97** | **63.04** | **139.31** | **73.12** | **13.71** | **0.00** |
| **Punctuation Percentage** | **6.76** | **3.09** | **5.54** | **3.19** | **7.30** | **0.00** |
| **Average Word Length** | **6.49** | **1.54** | **5.71** | **1.40** | **9.87** | **0.00** |
| **Word Count** | **31.09** | **13.267** | **25.46** | **16.05** | **7.23** | **0.00** |
| **Character Count** | **209.06** | **74.59** | **153.76** | **88.511** | **12.75** | **0.00** |
| **Hashtag Count** | **1.37** | **2.26** | **0.28** | **0.88** | **11.49** | **0.00** |
| **Numeric Count** | **0.21** | **0.51** | **0.06** | **0.26** | **6.55** | **0.00** |
| Uppercase Word Count | 0.69 | 1.42 | 0.91 | 2.63 | 2.02 | 0.04 |

| **Supplementary table 4. Significant differences between neutral and misconception tweets for carer-defined features (Manually coded set (N=1,414))** | | | | |
| --- | --- | --- | --- | --- |
| ***Feature*** | ***Neutral Percentage (n = 637)*** | ***Negative Percentage (n = 777)*** | ***Chi Square*** | ***p*** |
| **Senile** | **0.31%** | **30.89%** | **230.66** | **0.00** |
| **Demented** | **0.16%** | **46.98%** | **399.95** | **0.00** |
| **Donald Trump** | **0.00%** | **11.97%** | **81.61** | **0.00** |
| Nancy Pelosi | 0.00% | 1.16% | 7.43 | 0.01 |
| Deranged | 0.00% | 0.64% | 4.11 | 0.04 |
| **Memory** | **2.51%** | **0.13%** | **16.74** | **0.00** |
| Café | 0.31% | 0.00% | 2.44 | 0.12 |
| Insane | 0.16% | 0.77% | 2.69 | 0.10 |
| **Research** | **6.44%** | **0.13%** | **48.32** | **0.00** |
| Imbecile | 0.00% | 0.26% | 1.64 | 0.20 |
| Loon | 0.00% | 0.77% | 4.94 | 0.03 |
| **Crazy** | **0.00%** | **1.67%** | **10.76** | **0.00** |
| Looney Bin | 1.73% | 1.54% | 0.07 | 0.79 |
| Lunatic | 0.00% | 0.51% | 3.29 | 0.07 |
| Unhinged | 0.00% | 0.51% | 3.29 | 0.07 |
| **Senility** | **0.00%** | **3.35%** | **21.72** | **0.00** |
| **A weblink (URL)** | **68.92%** | **10.42%** | **515.07** | **0.00** |
| **Caregiver** | **2.04%** | **0.00%** | **16.00** | **0.00** |

| **Supplementary table 5. Comparison of significance found with manual testing and the ‘sklearn’ algorithm** | | |
| --- | --- | --- |
| *Feature* | *Significant with algorithm from ‘sklearn’* | *Significant from manual testing* |
| **Sentiment** | **Yes** | **Yes** |
| **Subjectivity** | **No** | **No** |
| **Body Length** | **Yes** | **Yes** |
| **Punctuation Percentage** | **Yes** | **Yes** |
| **Average Word Length** | **Yes** | **Yes** |
| Word Count | No | Yes |
| **Character Count** | **Yes** | **Yes** |
| **Hashtag Count** | **Yes** | **Yes** |
| **Numeric Count** | **Yes** | **Yes** |
| **Uppercase Word Count** | **No** | **No** |
| **Senile** | **Yes** | **Yes** |
| **Demented** | **Yes** | **Yes** |
| **Donald Trump** | **Yes** | **Yes** |
| **Nancy Pelosi** | **No** | **No** |
| **Deranged** | **No** | **No** |
| **Memory** | **Yes** | **Yes** |
| **Café** | **No** | **No** |
| **Insane** | **No** | **No** |
| **Research** | **Yes** | **Yes** |
| **Imbecile** | **No** | **No** |
| **Loon** | **No** | **No** |
| **Crazy** | **Yes** | **Yes** |
| **Looney Bin** | **No** | **No** |
| **Lunatic** | **No** | **No** |
| **Unhinged** | **No** | **No** |
| **Senility** | **Yes** | **Yes** |
| **A weblink (URL)** | **Yes** | **Yes** |
| **Caregiver** | **Yes** | **Yes** |

### 2.3 Features of Tweets from Campaign Period

| **Supplementary table 6. Significant differences between neutral and misconception tweets for literature-defined features (2^nd^ batch of extracted tweets based in the UK in the 6-month period surrounding our campaign (n = 7,124)) Corrected P value = 0.00625** | | | | | | |
| --- | --- | --- | --- | --- | --- | --- |
|  | Neutral | | Misconception | |  |  |
| Feature | Mean | Standard Deviation | Mean | Standard Deviation | *t* | *p* |
| **Sentiment** | **0.1**4 | **0.29** | **-0.03** | **0.28** | **25.72** | **0.00** |
| **Body Length** | **183.77** | **65.18** | **115.72** | **75.29** | **38.73** | **0.00** |
| Punctuation Percentage | 5..40 | 2.56 | 5.50 | 3.31 | 1.36 | 0.18 |
| Average Word Length | 5.72 | 1.31 | 5.68 | 1.53 | 1.09 | 0.81 |
| **Word Count** | **33.33** | **12.85** | **21.90** | **14.50** | **33.56** | **0.00** |
| **Character Count** | **216.10** | **76.12** | **136.62** | **88.86** | **38.46** | **0.00** |
| **Hashtag Count** | **0.9**7 | **2.**23 | **0.10** | **0.46** | **25.21** | **0.00** |
| **Numeric Count** | **0.23** | **0.**60 | **0.08** | **0.33** | **13.56** | **0.00** |

| **Supplementary table 7. Significant differences between neutral and misconception tweets for carer-defined features (2^nd^ batch of extracted tweets based in the UK in the 6-month period surrounding our campaign (n = 7,124)) Corrected P value = 0.0055556** | | | | |
| --- | --- | --- | --- | --- |
| ***Feature*** | ***Neutral Percentage (n = 4,470)*** | ***Misconception Percentage (n = 2,654)*** | ***Chi Square*** | ***p*** |
| **Senile** | **0.00%** | **13.60%** | **640.47** | **0.00** |
| **Demented** | **0.00%** | **22.00%** | **1071.433** | **0.00** |
| **Donald Trump** | **0.16%** | **4.56%** | **182.93** | **0.00** |
| **Memory** | **1.66%** | **0.08%** | **39.39** | **0.00** |
| **Research** | **3.78%** | **0.04%** | **100.16** | **0.00** |
| Crazy | 0.13% | 0.41% | 5.49 | 0.02 |
| **Senility** | **0.00%** | **2.03%** | **91.64** | **0.00** |
| **A weblink (URL)** | **50.92%** | **18.46%** | **773.52** | **0.00** |
| Caregiver | 0.13% | 0.00% | 3.57 | 0.06 |

## References

1. Delahanty, R., Alverez, J., Flynn, L. M., Sherwin, R. L., & Jones, S. S. (2019) Development and Evaluation of a Machine Learning Model for the Early Identification of Patients at Risk for Sepsis. *Annals of Emergency Medicine, 73*(4), 334-344 <https://doi.org/10.1016/j.annemergmed.2018.11.036>.
2. Neppalli, V. K., Medeiros, M., Caragea, C., Caragea, D., Tapia, A., & Halse, S. (2016).Retweetability analysis and prediction during Hurricane Sandy. *Proceedings of the ISCRAM 2016 Conference.* <https://www.cs.uic.edu/~cornelia/papers/iscram16b.pdf>
3. Oscar, N. Fox, P. A., Croucher, R., Wernick, R., Keune, J., & Hooker, K. (2017). Machine learning, sentiment analysis, and tweets: An examination of Alzheimer’s disease stigma on Twitter. *The Journals of Gerontology. Series B, Psychological Sciences and Social Sciences.* *72*(5), 742–751. <https://doi.org/10.1093/geronb/gbx014>.
4. Gretry, A., Davis, S. W., Horvath, C., & Belei, N. (2017). How tweet readability and brand hedonism affect consumer engagement. *Association for Consumer Research,* **45,** 629-633. <https://www.acrwebsite.org/volumes/v45/acr_vol45_1023601.pdf>
5. Farías, D. I. H., Patti, V., & Rosso, P. (2016). Irony detection in twitter: The role of affective content. *ACM Transactions on Internet Technology, 16*(3), 19:1 – 19:24. <https://dl.acm.org/doi/pdf/10.1145/2930663?casa_token=XtKc8CEol3AAAAAA:VRr1gRtQ3ehHIikMmZxRaaYOhiuVsHLVz1_G9uH0dsyZ1ptFyuZx1oyk3fH6Sn-TDTO4Jq9BToYL>
6. Rosen, A., & Ihara, I. Giving you more characters to express yourself. Twitter. (2017). <https://blog.twitter.com/official/en_us/topics/product/2017/Giving-you-more-characters-to-express-yourself.html>
7. Salton, G., and McGill, M. *Introduction to Modern Information Retrieval.* (1986). McGraw-Hill, Inc, New York.
